# EGCG Enhanced the Anti-tumor Effect of Doxorubicine in Bladder Cancer via NF-κB/MDM2/p53 Pathway

**DOI:** 10.3389/fcell.2020.606123

**Published:** 2020-12-23

**Authors:** Ke-Wang Luo, Xiao-hong Zhu, Ting Zhao, Jin Zhong, Han-chao Gao, Xin-Le Luo, Wei-Ren Huang

**Affiliations:** ^1^Key Laboratory, People's Hospital of Longhua, The Affiliated Hospital of Southern Medical University, Shenzhen, China; ^2^Key Laboratory of Medical Programming Technology, Shenzhen Second People's Hospital, The First Affiliated Hospital of Shenzhen University, Shenzhen, China; ^3^Department of Nephrology, Shenzhen Longhua District Central Hospital, Affiliated Central Hospital of Shenzhen Longhua District, Guangdong Medical University, Shenzhen, China

**Keywords:** MDM2, p53, NF-κB, bladder cancer, doxorubicine, EGCG

## Abstract

Doxorubicin (DOX), the first-line chemotherapy for bladder cancer, usually induces side effects. We previously demonstrated that green tea polyphenol EGCG had potent anti-tumor effect in bladder cancer via down regulation of NF-κB. This study aimed to investigate the additive/synergistic effect EGCG and DOX against bladder cancer. Our results demonstrated that the combined use of DOX and EGCG inhibited T24 and SW780 cell proliferation. EGCG enhanced the apoptosis induction effect of DOX in both SW780 and T24 cells and resulted in significant differences. Besides, EGCG promoted the inhibitory effect of DOX against bladder cancer cell migration. In addition, the *in vivo* results demonstrated that DOX in combination with EGCG showed the most potent anti-tumor effects among DOX, EGCG and DOX+EGCG treatment groups. Further mechanistic studies determined that the combination of DOX and EGCG inhibited phosphorylated NF-κB and MDM2 expression, and up-regulated p53 expression in tumor, as assessed by western blot and immunohistochemistry. Western blot in SW780 cells also confirmed that the combined use of EGCG and DOX caused significant increase in p53, p21, and cleaved-PARP expression, and induced significant inhibition in phosphorylated NF-κB and MDM2. When NF-κB was inhibited, the expression of p53 and p-MDM2 were changed, and the combination of DOX and EGCG showed no obvious effect in transwell migration and cell viability. In conclusion, the novel application of chemotherapy DOX and EGCG demonstrated potent anti-tumor, anti-migration and anti-proliferation effects against bladder cancer. EGCG enhanced the anti-tumor effect of DOX in bladder cancer via NF-κB/MDM2/p53 pathway, suggesting the potential clinical application against bladder cancer patients.

## Introduction

Despite significant advances in cancer research, cancer remains a worldwide health problem, with an estimated 9.6 million deaths in 2018. Bladder cancer ranks 9th of incidence and caused about 430,000 new cases annually worldwide (Antoni et al., 2017). It is estimated that ~549,939 persons are diagnosed with bladder cancer worldwide each year (Stewart and Wild, 2014). Therefore, novel therapeutic strategies and more effective combinations for bladder cancer treatment are still needed.

Doxorubicin (DOX), a first-line chemotherapy for bladder cancer, inhibits cell proliferation by directly interfering with DNA synthesis and transcription, with a broad anti-tumor spectrum (Chen et al., 2018). It is reported that DOX could regulate the expression of p53 to exert its antitumor effect. Lu et al. found that DOX increased p53 expression in A549 cells, and thereby promoted cell apoptosis and necrosis (Lu et al., 2014). Doxorubicin was demonstrated to be effective in induction of apoptosis in prostate cancer, by activating p53-mediated pathway to exert their anti-cancer effects by causing DNA damage and initiating cell cycle arrest in cancer cells (Yang et al., 2016). Though DOX was widely used in the treatment of bladder and other cancers, the drug could cause immune function declines and induce severe cardiac toxicity with long-term application (Renu et al., 2018). Therefore, it is of great importance to find combined treatment plans with synergistic antitumor efficacy or reducing the side effects of doxorubicin.

Epigallocatechin-3-gallate (EGCG), the most bioactive polyphenol from *Camellia sinensis*, has been demonstrated to have various biological activities, including anti-oxidation, anti-cancer and others (Gan et al., 2018). EGCG was found to be effective in improving liver and pancreatic β-cell functions in iron-overloaded β-thalassemia mice by diminishing redox iron and free radicals (Koonyosying et al., 2018). Besides, numerious literatures also reported the anti-cancer effect of EGCG. It was demonstrated that tea polyphenol EGCG was effective in decreasing the risk of several cancer types, including stomach, prostate, and lung cancers (Lin and Liang, 2000). Treatment with EGCG resulted in significant down-regulation of tumor burden in breast, bladder, and prostate cancers (Yang et al., 2009). EGCG was also clarified to be effective in inhibiting metastasis or angiogenesis, or promoting apoptosis in leukemia, breast, prostate cancer cells (Gan et al., 2018). We previously found that treatment with green tea aqueous extract resulted in obviously inhibition of 4T1 breast tumor by induction of apoptosis, and EGCG was shown to be the most abundant ingredient in green tea (Luo et al., 2014). We also demonstrated that EGCG suppressed the bladder cancer SW780 tumor growth by down regulation of NF-κB (Luo et al., 2017). Recently, varieties of studies investigated the effects of EGCG in combination with chemotherapies as adjuvant agent. Wang et al. found that EGCG enhanced cisplatin sensitivity and increased the drug accumulation of cisplatin in ovary cancer by regulating expression of the copper and cisplatin influx transporter CTR1 (Wang et al., 2015); Chen et al. determined that EGCG synergistically enhanced DOX anticancer effects involving autophagy inhibition in hepatocellular carcinoma as a chemotherapeutic augmenter (Chen et al., 2014). EGCG is naturally derived and can be used as a preventive or sensitizer in the prevention and treatment of cancer, and could inhibit bladder cancer proliferation and migration by down regulation of NF-κB. DOX is a first-line chemotherapy drug for bladder cancer, but usually induces side effects. This study aims to investigate the synergistic/additive anti-tumor effect of DOX and EGCG in bladder cancer and determine the underlying mechanisms, when DOX in a much lower dose than clinic use.

During the process of cancer propagation, Murine double minute-2 (MDM2) plays an important role, which serves as a negative regulator of p53 and thereby limits cell cycle arrest and apoptosis (Zhao et al., 2018). It is reported that MDM2 can bind to p53 and block the p53 signaling pathway, and could also promote the degradation of p53; when p53 is activated, in turn, it also inhibits the transcriptional expression of MDM2 (Thomasova et al., 2012). Besides, NF-κB can promote the expression of oncogene MDM2, which in turn inhibits the activation of p53 (Zhuang et al., 2014). We previously found that EGCG was effective in down-regulated the expression of NF-κB and inhibited bladder tumor growth (Luo et al., 2017); while DOX could regulate the expression of p53 to exert its antitumor effect. Thus, we hypothesize that tea polyphenol EGCG could work synergistically with DOX in inhibiting bladder cancer cells proliferation and migration via NF-κB/MDM2/p53 pathway.

The present study aimed to investigate the combined use of DOX and EGCG against tumor growth, proliferation, and migration in bladder cancer. We hypothesize that tea polyphenol EGCG can work synergistically with DOX in inhibiting bladder cancer cells proliferation and migration. Here, we assessed the apoptosis-induction and anti-migration abilities of the combination of DOX and EGCG *in vitro*, and then further evaluated the anti-tumor activities of DOX and EGCG in mice *xenograft* model. Besides, the protein expression of NF-κB, p53, and MDM2 were also evaluated both *in vitro* and *in vivo* after treatment. Immunohistochemistry and western blot were used to determine the underlying mechanism of anti-metastasis activities of DOX+EGCG.

## Materials and Methods

### Cells and Reagents

Bladder cancer SW780 and T24 cells were cultured in DMEM medium containing 10% (v/v) fetal bovine serum and 1% penicillin-streptomycin (Life technology, United States) at 37°C in 5% CO_2_ humidified incubator. MTT and Doxrubicine (DOX) were obtained from Sigma, USA. Transwell plates were from Corning Incorporated, United States. Annexin V-FITC kit was purchased from BD Pharmingen, United States. NF-κB, NF-κB p65 (p-NF-κB), MDM2, Phospho-MDM2 (p-MDM2), p53, p21, Bcl-2, and PARP were purchased from Cell signaling technology, United States. Alanine transaminase (ALT), Creatine kinase (CK), and aspartate transaminase (AST) kits were obtained from Stanbio, United States. NF-κB inhibitor (SC75741) was purchased from Selleck, United States.

### Cell Viability Assay

Cells (1 × 10^4^/well) were seeded in 96-well plates (Corning, United States) and incubated with DOX (0, 0.05, 0.1, 0.2, 0.4, and 0.8 μM) and EGCG (0, 12.5, 25 μM) for 48 h. Following incubation, MTT (30 μl/well) was added and the plate was incubated for additional 4 h. Then, the absorbance of each sample was measured at 540 nm as a reference with the microplate reader (Thermo Multiskan GO, United States).

### Annexin V Double Staining

SW780 cells were treated with EGCG (100 μM) and/or DOX (0.1 μM) for 24 h, and T24 cells were treated with EGCG (200 μM) and/or DOX (0.1 μM) for 24 h. Then, the cells were harvested and washed with cold PBS. After stained with Annexin V-FITC and PerCP for 15 min in dark at room temperature, the fluorescent signal was assessed by flow cytometry (FACSARIA II, Becton Dickinson). Positioning of quadrants on annexin-V plots was performed to distinguish living cells (FITC–/PerCP–), early apoptotic cells (FITC+/PerCP–), and late apoptotic or necrotic cells (FITC+/PerCP+).

### Cell Migration Assays

The efficacy of EGCG and/or DOX against SW780 and T24 cell migration *in vitro* were assessed by scratch wound healing and transwell migration assays.

In scratch wound healing assay, cells (1 × 10^5^/well) were seeded in 24-well plates. After incubated in FBS-free medium for 24 h, cells were scraped with crosses using yellow pipette tips, and then added with full medium (with 10% FBS) with EGCG and/or DOX. SW780 cells were treated with EGCG (12.5 μM) and/or DOX (0.1 μM) for 24 h, and T24 cells were treated with EGCG (25 μM) and/or DOX (0.1 μM) for 24 h. Then each well was photographed under a microscope (Olympus IX73). The percentages of open wound area were measured and calculated using the TScratch software.

During transwell migration assay, cells (2 × 10^4^/well) were added into transwell upper chambers with 100 μl medium containing EGCG and DOX (with 1% FBS). And the concentration of EGCG and DOX were the same as the dose in scratch wound assay. Then 500 μl full DMEM medium (with 10% FBS) was added in the lower chamber. Cells were allowed to migrate from the upper layer through the chamber membrane to the lower well for 24 h incubation at 37°C. Later, cells were fixed with 100% methanol and stained with 0.1% crystal violet for 10 min. Stained membranes were photographed using microscope (Olympus IX73), and the migrated rates were assessed by manual counting of the cells in chamber membrane.

### Nude Mice Tumor Model

BALB/c mice (female, 6–8 weeks) were purchased from Vital River Laboratory Animal Technology Co. Ltd, Beijing, and were kept under pathogen-free conditions in Guangdong Medical University. The animal experiments were carried out under the permission of the animal ethical and welfare committee. The sensitive cell line SW780 was chosen for animal study. Cells (3 × 10^6^ in 0.2 ml PBS) were subcutaneously (s.c.) inoculated at the back of mice. After the tumor size reached to 80–100 mm^3^, the mice were randomly grouped (*n* = 7): control group (saline, i.p., injected everyday), EGCG group (50 mg/kg EGCG, i.p., injected everyday), DOX group (2 mg/kg DOX, i.p., injected once) and EGCG+DOX group (50 mg/kg EGCG, i.p., injected everyday, and 2 mg/kg DOX, i.p., injected once). Treatments were initiated after the tumor size reached 80–100 mm^3^, and lasted for 3 weeks. During treatment, the body weight and tumor volume of each mouse was measured twice a week. At day 28, mice were sacrificed, and the tumors were removed for quantification of tumor burden and immunohistochemisty staining. The tumors were also cut up and lysed for protein analysis. The effect of treatments on hemato-biochemical markers were assessed by measuring the activities of liver or heart related enzymes (ALT, AST, and CK) in the plasma using assay kits purchased from Stanbio Co. Ltd.

### Western Blot Analysis

Tumors were cut into pieces, grinded, and lysed in lysis buffer. Cells treated with EGCG (100 μM) and/or DOX (0.1 μM) were also lysed in lysis buffer. After protein concentration measured, protein samples (20 μg) were electrophoresed in 10 % SDS-PAGE gel and transferred to PVDF membrane (Millipore, United States). After blocking with 10% non-fat milk, the membranes were washed with PBS-T, and then incubated with primary antibodies for 24 h at 4°C. After washing, the membrane was incubated with secondary antibodies for 1 h. Finally, visualization of protein bands was performed using the ECL substrate reagent kit (GE Healthcare) on a Gel Doc XR imaging system (Bio-RAD, United States).

### Immunohistochemistry

Tumor tissues were fixed with 10% formalin solution for 7 days at room temperature. Then the samples were paraffin embedded, sectioned longitudinally at 3 μm. After the sections dewaxing in xylene, 100, 95, 80, and 70% ethanol and running water for 1 min, respectively, the sections were blocked with BSA solution for 20 min. Then the sections were probed with primary antibody at 4°C overnight. After washing, the sections were incubated with secondary antibody for 1 h at room temperature. The sections were washed 3 times with PBS for 5 min each time, and incubated with streptavidin-biotin peroxidase at room temperature for 30 min. Finally, the sections were colored with DAB for 5 min, stained with hematoxylin and mounted. The stained sections were examined and photographed using an Olympus IX73 microscope (Japan).

### Statistical Analysis

All data were expressed as mean ± SD/SEM. Statistical analysis was performed using one way ANOVA, with *p* < 0.05 as regarded statistically significant.

## Results

### Effect of the Combined Use of EGCG and DOX on Bladder Cancer SW780 and T24 Cell Viability

**Figure 1:**
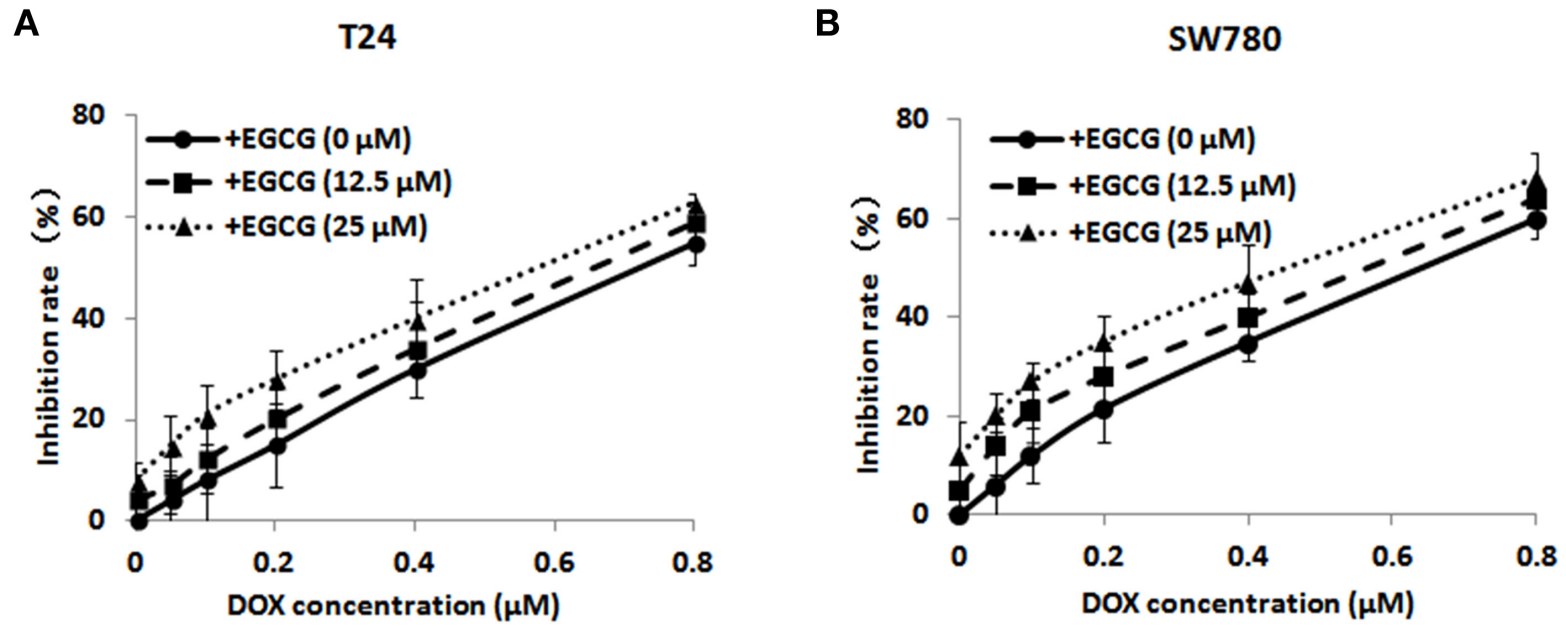
The effects of EGCG in combination of various doses of DOX on bladder cancer T24 **(A)** and SW780 **(B)** cell viability. Cells were incubated with EGCG (0, 12.5, and 25 μM) in the presence of various concentrations of EGCG (0, 0.05, 0.1, 0.2, 0.4, and 0.8 μM) after 48 h treatment. Data were expressed as mean ± SD (*n* = 3).

MTT assay was performed to assess the effect of EGCG in combination with DOX at different concentrations on cell viability of bladder cancer SW780 and T24 cells, and then the effective doses of DOX and EGCG would be chosen for further studies when the synergistic index (CI) was lower than 1. As shown in Figure 1, the combination of EGCG and DOX inhibited cell viability significantly. The inhibition rate of the combination of EGCG and DOX was higher than DOX alone at various concentrations. The combination of DOX and EGCG demonstrated additive or synergistic cytotoxic effects, especially at the doses of 0.1 μM DOX plus 25 μM EGCG in T24 cells, which produced an inhibition of 21.8% cell viability, while DOX and EGCG alone caused inhibition rates of 12.3 and 5.2 %, respectively. Besides, the combined use of DOX (0.1 μM) and EGCG (12.5 μM) on SW780 cells also demonstrated additive cytotoxic effects, which produced an inhibition of 22.1% cell viability, while DOX and EGCG alone caused inhibition rates of 12.1 and 8.6%, respectively. Since the combination of DOX (0.1 μM) and EGCG (12.5 μM, 25 μM) has the best additive/synergistic inhibition capacity of cell viability in SW780 and T24 cells, respectively, such dose of combination was selected in further studies.

### EGCG Enhanced the Apoptosis Induction Effect of DOX in SW780 and T24 Cells

**Figure 2:**
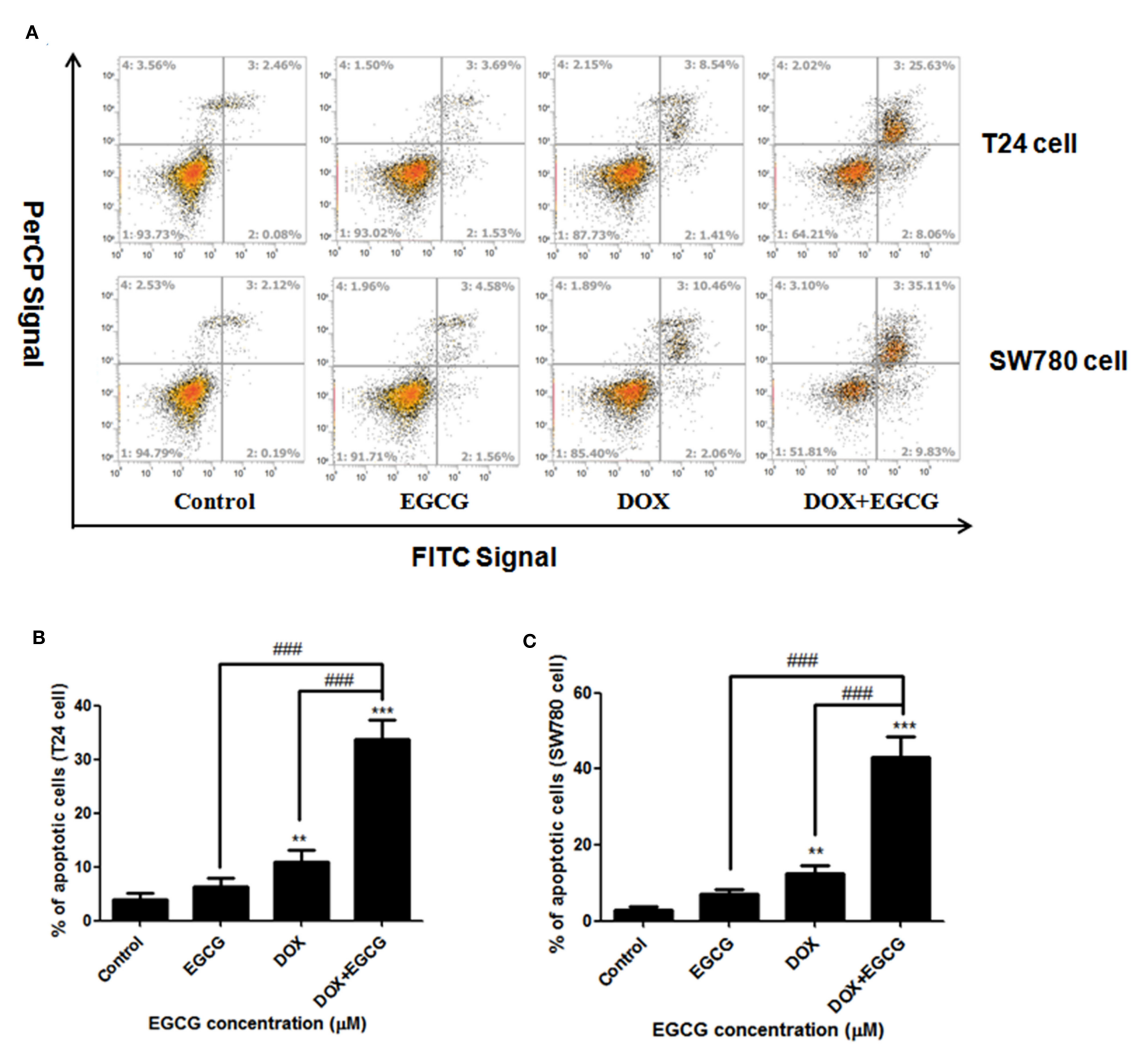
EGCG enhanced the apoptosis induction effect of DOX in SW780 and T24 cells. **(A)** Flow cytometry images. **(B)** Quantitative analysis of the percentage of apoptotic cells of EGCG and/or DOX on bladder cancer T24 cells **(B)** and SW780 cells **(C)**, after 24 h incubation. The percentage of total apoptotic cells was defined as the sum of early and late apoptotic cells. Data were presented as mean + SD (*n* = 3). **p < 0.01 and ***p < 0.001, as compared with untreated control. ###*p* < 0.001, as compared between groups indicated.

Annexin-V FITC/Per CP staining showed that treatments of EGCG, DOX, and EGCG plus DOX, resulted in significant apoptosis induction efficacies in bladder cancer SW780 and T24 cells (Figure 2). The percentage of apoptotic cells upon treatment with 0.1 μM of DOX was found to be 12.52 and 9.95% in SW780 and T24 cells, while DOX plus EGCG caused an increasing of 42.94 and 33.69% of apoptotic cells in SW780 and T24 cells, respectively. Significant differences were shown between DOX+EGCG and EGCG groups, and DOX+EGCG and DOX groups, indicating EGCG enhanced the apoptosis induction effect of DOX in bladder cancer.

### EGCG Promoted the Anti-migration Effect of DOX in SW780 and T24 Cells

**Figure 3:**
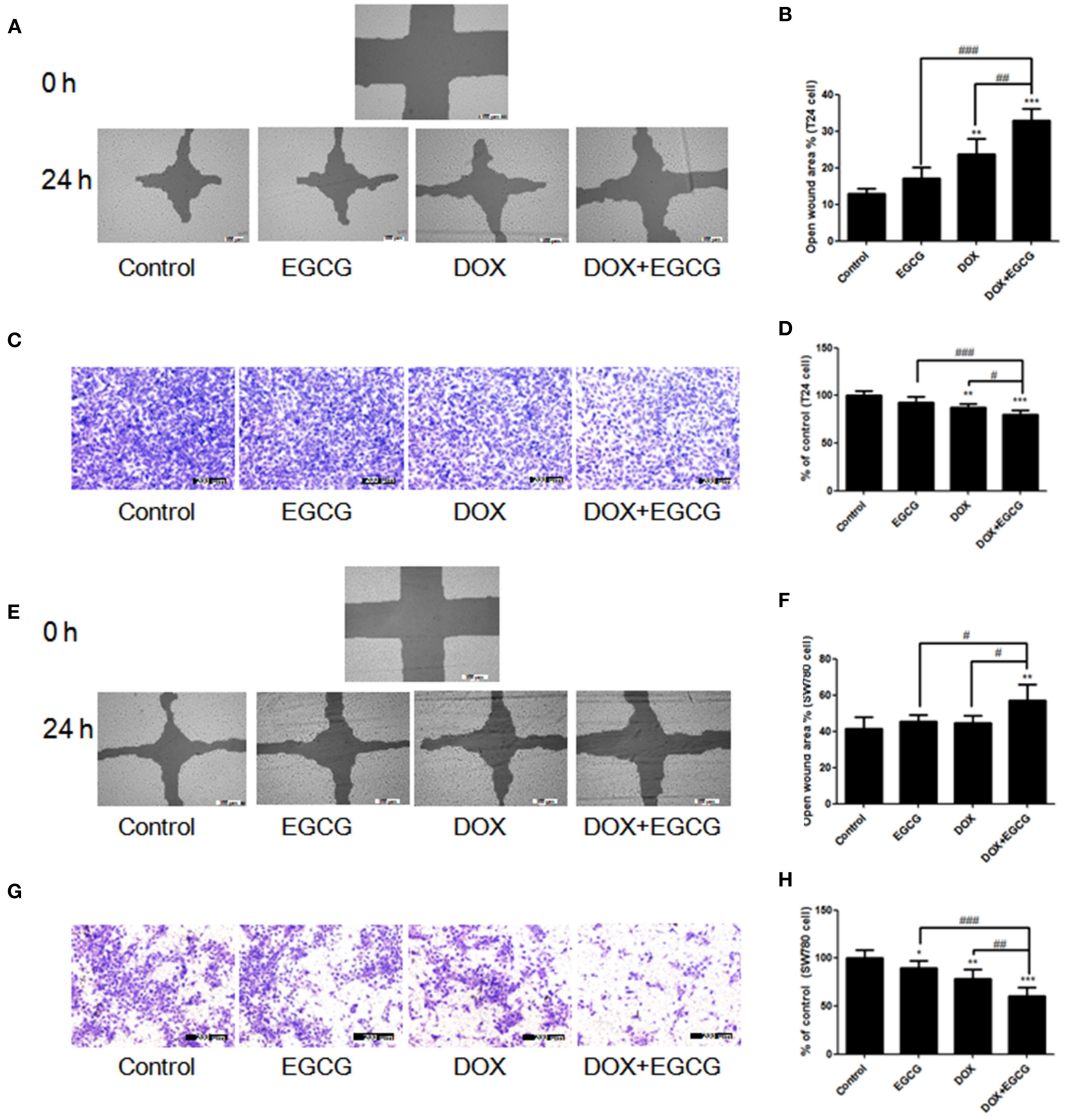
EGCG promoted the anti-migration effects of DOX in T24 and SW780 cells. The efficacy of EGCG and/or DOX against bladder cancer cell migration *in vitro* were assessed by scratch wound healing and transwell migration assays. **(A,E)** Representative images of the wounded cell monolayers of T24 cells **(A)** and SW780 cells **(E)**. **(B,F)** Quantitative analysis of the anti-migration activity of EGCG and/or DOX in T24 **(B)** and SW780 cells **(F)**, as assessed by scratch wound healing assay. Data were expressed as the percentage of open wound area from baseline cultures without treatment. **(C,G)** Representative images of the stained migrated T24 **(C)** and SW780 cells **(G)** in filters in transwell migration assay. **(D,H)** Quantitative analysis of the transwell migration activity of EGCG and/or DOX in T24 cells **(D)** and SW780 cells **(H)**. The migrated cells were quantified by manual counting and represented as a percentage of control values. Data were presented as mean + SD (*n* = 3). *p < 0.05, **p < 0.01, and ***p < 0.001, as compared with untreated control. #*p* < 0.05, ##*p* < 0.01, and ###*p* < 0.001, as compared between groups indicated.

To determine the efficacy of EGCG and/or DOX against cancer cell migration *in vitro*, the scratch wound healing and transwell migration assays were introduced. As shown in Figure 3A, treatment of EGCG at 25 μM resulted in no obvious effect in inhibition of T24 cell migration and invasion, and DOX (0.1 μM) alone induced significant decrease of T24 cell metastasis. The inhibition effect of DOX in cell migration and invasion was enlarged when in combination with EGCG, and significant differences were shown between DOX+EGCG and DOX (Figures 3C,D). Besides, similar results were also shown in SW780 cell migration and invasion when treated with EGCG, DOX and DOX+EGCG. In Figure 3F, no significant difference was shown in EGCG and DOX treatment alone in SW780 cell migration, and the combination of EGCG and DOX caused significant increase of open wound area, indicating that DOX+EGCG treatment demonstrated synergistic effect in SW780 cell migration. Furthermore, the combined use of EGCG and DOX showed better effect in SW780 cell invasion than individual treatment, the cell invasion was inhibited to 56.2%, and significantly differences were shown to individual treatments (Figure 3H).

### EGCG Enhanced the Anti-tumor Effect of DOX in Nude Mice Tumor Model

**Figure 4:**
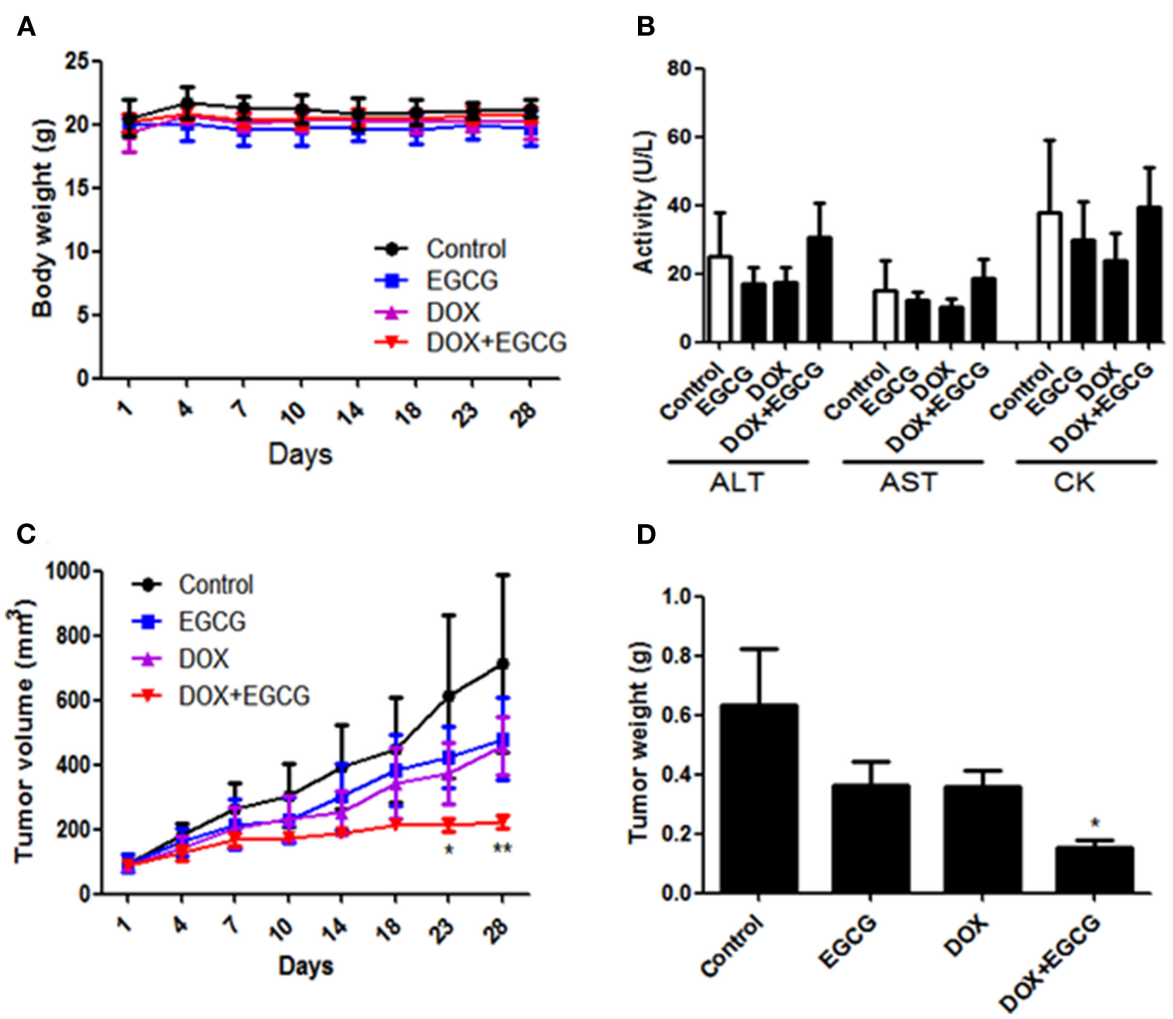
EGCG enhanced the anti-tumor effect of DOX in SW780 nude mice xenograft tumor model. **(A)** No significant body weight loss of mice was found during EGCG treatment period. **(B)** Evaluation of the hemato-biochemical markers (ALT, AST, and CK) of mice plasma after treated with EGCG and/or DOX. **(C)** Quantitative analysis of the tumor volume in each group during treatment. Tumor volume was assessed by caliper and calculated as the length * width * width * 0.5. The combination of DOX+EGCG demonstrated significant inhibition of tumor volume from day 23, when compared with untreated control. **(D)** Graph showed the tumor weight from different group. Data were expressed as mean +/ ± SEM, *n* = 7. *p < 0.05 and **p < 0.01, as compared with control.

To evaluate the activity of EGCG and/or DOX on tumor growth *in vivo*, a subcutaneous tumor model in nude mice was employed. No significant body weight loss was found in all treatment groups (Figure 4A). Besides, hemato-biochemical markers test showed that no obvious difference was shown on plasma activities of heart specific (CK) and liver related (AST and ALT) enzymes between untreated control and treatment groups (Figure 4B). As shown in Figure 4C, the tumor volume were decreased in EGCG and DOX individual treatment group, however no significant difference was shown. The combination of EGCG and DOX demonstrated significant inhibition of tumor volume from day 23, when compared with untreated control. In addition, tumor weights were decreased in all treatment groups, and significant difference was only shown in DOX+EGCG treatment group (by 76.8%) when compared with control (Figure 4D). These results clarified that EGCG enhanced the anti-tumor effect of DOX in bladder cancer, and the combination of DOX and EGCG showed the best anti-tumor effects among the treatment groups.

### The Combination of EGCG and DOX Inhibited NF-κB, p53, and MDM2 Expression in Tumor

**Figure 5:**
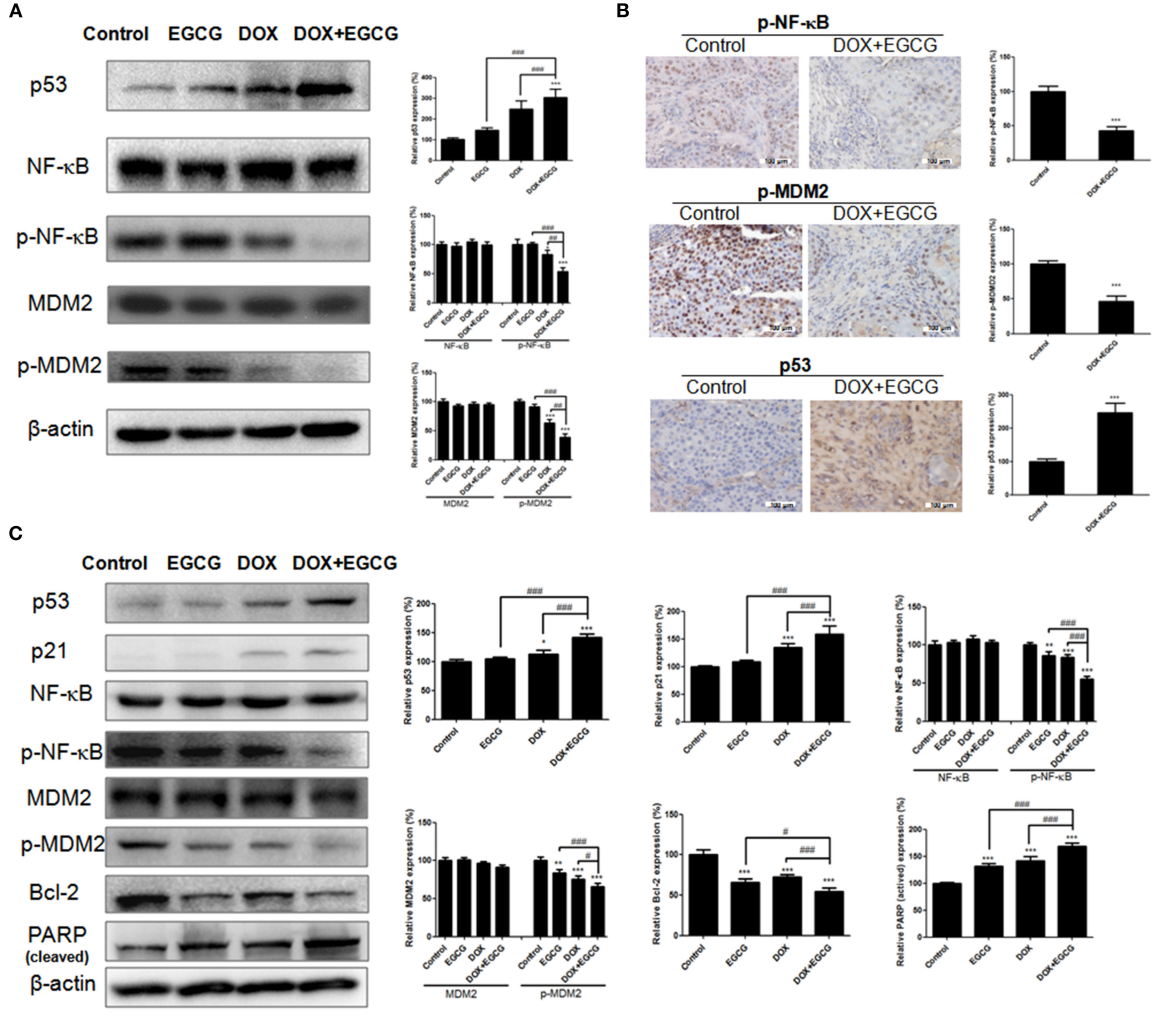
Effects of DOX and/or EGCG on protein expressions in tumor and in SW780 cells. **(A)** Representative images of protein expressions and statistical analysis of p53, NF-κB, p-NF-κB, MDM2, and p-MDM2 expressions in SW780 tumors after treatment. **(B)** Representative immunohistochemical images and statistical analysis of p53, p-NF-κB, and p-MDM2 expressions in SW780 tumors as assessed by immunohistochemistry. Data were expressed as mean + SD, *n* = 3. ***p < 0.001, as compared with control. ##*p* < 0.01 and ###*p* < 0.001, as compared between groups indicated. **(C)** Representative images of protein expressions and statistical analysis of p53, p21, NF-κB, p-NF-κB, MDM2, p-MDM2, Bcl-2, and PARP expressions in SW780 cells. Data were showed as mean + SD (*n* = 3). *p < 0.05, **p < 0.01, and ***p < 0.001, as compared with untreated control. #*p* < 0.05 and ###*p* < 0.001, as compared between groups indicated.

In order to evaluate the protein expression of NF-κB, p53, and MDM2 in tumor, the western blot and immunohistochemistry were performed. As shown in Figure 5A, treatment of EGCG, DOX, and DOX+EGCG in nude mice resulted in obviously increase in p53, and inhibition of phosphorylated NF-κB and MDM2 in protein. Semi-quantitative analysis demonstrated that EGCG promoted the effect of DOX in increase of p53 expression and decrease of p-NF-κB and p-MDM2 expression, and the combination DOX+EGCG showed the best result among treatment groups. Significant differences were shown between DOX+EGCG and individual treatments. In addition, similar results were also shown in immunohistochemistry that p-NF-κB and p-MDM2 were down regulated, and p53 was up regulated in DOX+EGCG group, and significant differences were shown when compared with control (Figure 5B).

### EGCG and DOX Regulated the Protein Expressions

In order to evaluate the expression of NF-κB, p53, MDM2, and other related proteins in bladder cancer SW780 cells, the western blot was performed. As shown in Figure 5C, EGCG treatment induced no obvious effect in p53 and p21 expression, while the combined use of EGCG and DOX caused significant increase in p53 and p21 expression, and significant differences were shown to EGCG and DOX individual treatments. There was no obvious change shown in NF-κB and MDM2 expression among treatment groups, however the activated phosphorylated NF-κB and phosphorylated MDM2 was down-regulated significantly in EGCG and DOX individual groups, and the combination group of DOX+EGCG enlarged the inhibition and showed the best result among three treatment groups. Besides, EGCG, DOX and DOX+EGCG treatment resulted in significant decreasing in Bcl-2 expression, and significant increase of cleaved-PARP, indicating the activation of apoptosis.

### The Combination of EGCG and DOX Showed no Obvious Effect on Transwell Migration and Cell Viability When NF-κB Was Inhibited

**Figure 6:**
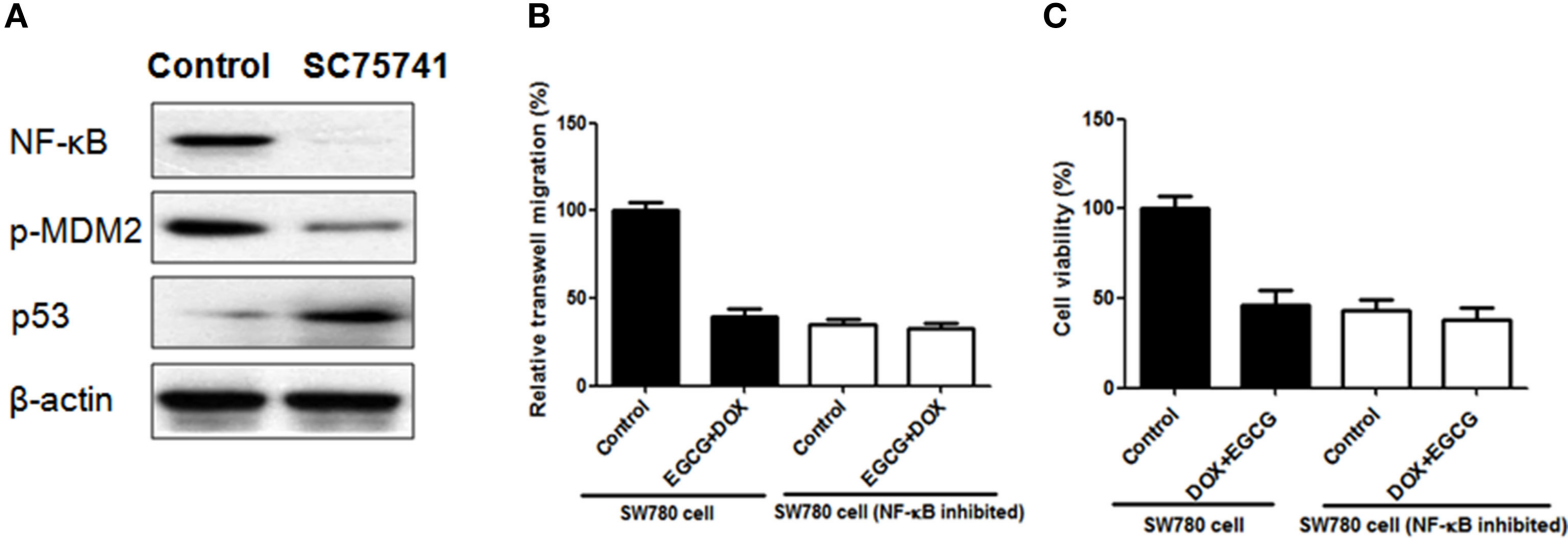
Effects of DOX+EGCG on transwell migration and cell viability when NF-κB was inhibited. **(A)** Protein expression of NF-κB, p-MDM2, and p53, when SW780 treated with SC75741 (NF-κB inhibitor). Anti-migration **(B)** and anti-proliferation **(C)** effects of DOX+EGCG in normal and NF-κB inhibited SW780 cells. Data were expressed as mean + SD, *n* = 3.

In order to confirm the importance of NF-κB and NF-κB/MDM2/p53 pathway in EGCG+DOX induced migration and prolifertaion inhibition, SC75741 (NF-κB inhibitor) was added in SW780 cells, and then the cells were harvested for transwell and MTT assays. As shown in Figure 6A, when SC75741 was added, the NF-κB was blocked, and expression of p-MDM2 was decreased and p53 was up-regulated. Besides, the combination of DOX+EGCG showed no obvious effect in transwell migration when NF-κB inhibited, which worked effectively in normal SW780 cells. The cell viability analysis in Figure 6C showed similar results that DOX+EGCG treatment led to no difference in cell viability in NF-κB inhibited SW780 cells, when compared with control.

## Discussion

Green tea is the most popular beverage consumed worldwide, and EGCG is the most abundant and bioactive tea Polyphenol. EGCG has been widely consumed as health-promoting food ingredients and showed chemotherapeutic efficacies in cancer prevention and treatment. In this study, we aim to investigate the additive/synergistic effect of EGCG in combination with DOX against bladder cancer both *in vitro* and *in vivo*.

In the present study, we demonstrated that treatment of EGCG in combination with DOX resulted in dose-dependent inhibition of cell viability in T24 and SW780 cells *in vitro* (Figure 1). The combination of DOX (0.1 μM) and EGCG (12.5 μM, 25 μM) has the best additive/synergistic inhibition effect of cell viability in SW780 and T24 cells, respectively, and these dose of combination was selected in further studies. In order to determine whether the anti-proliferative effect of EGCG and DOX was associated with apoptosis induction, Annexin V assay was employed. The results showed that DOX plus EGCG caused significant increases of percentage of apoptotic cells in bladder cancer T24 and SW780 cells, when compared to the individual treatment of DOX or EGCG (Figure 2), indicating the synergistic effect of EGCG to DOX on apoptosis induction. The findings were in line with the results in head and neck cancer that EGCG exhibited synergistic apoptosis is induction effects in combination with chemotherapy erlotinib (Haque et al., 2015). EGCG was also demonstrated to be effective in reducing cell viability and migration in oral cancer when combined with 5-fluorouracil (Pons-Fuster López et al., 2019). In addition, the combination of DOX and EGCG showed greater level inhibition of cell migration on T24 and SW780 cells, when compared with individual treatment of DOX or EGCG, as assessed by scratch wound healing and transwell migration assays (Figure 3). These results indicated that EGCG enhanced the inhibitory effect of DOX on cell migration in bladder cancer T24 and SW780 cells, which was in line with Flores-Pérez's finding that EGCG enhanced cisplatin-induced inhibition in cell migration and sensitize A549 cells to chemotherapy (Flores-Pérez et al., 2016). Moreover, the selected does of EGCG and DOX for migration and invasion assays were non-cytotoxic doses, and the cell viability was about 80%. This non-cytotoxic does of DOX+EGCG showed remarkable anti-migration effects to bladder cancer cells, revealing that EGCG could be added as an effective supplement for bladder cancer prevention and treatment.

In addition to *in vitro* studies, the *in vivo* anti-tumor effects of DOX and EGCG were evaluated in a xenograft mouse model. No obvious toxicity was shown to the mice after treatment, as assessed by the body weight and hemato-biochemical markers (AST, ALT, and CK). Administration of EGCG (50 mg/kg) and DOX (2 mg/kg) was able to decrease the tumor volume and tumor weight in mice bearing SW780 tumors. The combination of EGCG and DOX demonstrated significant inhibition of tumor volume from day 23, and also caused significant inhibition of tumor weight (Figures 4C,D). The finding suggested that DOX and EGCG could work together against tumor growth, and showed better anti-tumor effect when compared with DOX or EGCG alone. It is demonstrated that EGCG/gelatin-doxorubicin gold nanoparticles enhanced the therapeutic efficacy of doxorubicin for prostate cancer treatment (Tsai et al., 2016). A recent preclinical study revealed that EGCG attenuated DOX-induced cardiotoxicity in rats (Saeed et al., 2015). Besides, reports also showed that EGCG could work as an adjuvant in cancer treatment to enhance the efficacy of chemotherapy, such as EGCG inhibited DNA repair and enhanced cisplatin sensitivity in human cancer cells both *in vitro* and *in vivo* (Heyza et al., 2018), and the 5-FU and EGCG co-loaded nanoparticles could sustained drug release, and enhanced cellular uptake, thus exhibited superior anti-tumor activity and pro-apoptotic efficacy *in vivo* against colon cancer (Wang et al., 2019).

In our study, the *in vivo* doses of EGCG and DOX were 50 and 2 mg/kg, respectively, which were based on the clinical dose of DOX and our previous study of EGCG. The clinical dose of DOX on bladder cancer patient was about 60 mg for a 60 kg adult per month, which was equivalent to a single dose of 12.3 mg/kg for mouse per month. We chose the dose of 2 mg/kg of DOX in combination study *in vivo*, which was much lower than the clinical dose transfer to mouse, and would not induce severe side effects. Besides, we previously found that EGCG at 100 mg/kg (i.p., injected daily) decreased the tumor volume by 68.4% in mice bearing SW780 tumors, while EGCG at the dose of 50 mg/kg showed no obvious inhibition in bladder tumor (Luo et al., 2017). In order to investigate the additive/synergistic effect of EGCG and DOX, we adopt the ineffective dose of EGCG (50 mg/kg) and much lower than clinical dose of DOX (2 mg/kg) in the combination study. Thus, if the combination was work, it means the ineffective dose of EGCG plus DOX could work synergistically, and enhance the anti-tumor effect of DOX. On the other hand, the dose of DOX (2 mg/kg) was much lower than clinical dose transferred to mouse, and the unwanted side effects could be lowered. The *in vivo* animal study demonstrated that the combination of EGCG and DOX showed significant inhibition in bladder tumor, while the individual treatments exhibited no obvious effect. That indicates EGCG can synergistically enhance the action of DOX in inhibiting the bladder tumor growth, as adjuvant therapy in bladder cancer treatment.

To gain insight into the underlying mechanism of DOX+EGCG induced anti-tumor efficacies, western blot and immunohistochemistry were employed. As shown in Figure 5C, treatment of DOX+EGCG in nude mice resulted in significant increase in p53, and obvious inhibition of phosphorylated NF-κB and MDM2, with significant differences to individual treatment of EGCG or DOX. Similar results were also shown in immunohistochemistry analysis that the combination of DOX and EGCG was effective in down-regulation of p-NF-κB and p-MDM2, and up-regulation of p53 (Figure 5B). The *in vivo* animal results and protein analysis in tumor indicated that the combination of DOX and EGCG showed the best anti-tumor effect and the strongest protein regulation in p53, p-NF-κB, and p-MDM2, among three treatment groups of DOX, EGCG, and DOX+EGCG. In addition, protein expressions of p53, p21, NF-κB, MDM2, PARP, and Bcl-2 were also tested in SW780 cells. As shown in Figure 5C, DOX was effective in up-regulation of p53 and p21, and the increases were enlarged when DOX combined with EGCG, with significant difference to DOX. Treatment of DOX alone resulted in activation of NF-κB and MDM2, and the combination of DOX+EGCG caused strongest decrease on p-NF-κB and p-MDM2 among three treatment groups. Similar results were also found on the effect of PARP and Bcl-2, DOX+EGCG showed better effect than DOX treatment alone. The result was in agreement with Pan's finding that treatment of mice with EGCG markedly attenuated cisplatin induced mitochondrial oxidative stress and damages through decreasing NF-κB (Pan et al., 2015). Besides, the *in vitro* findings in proteins were completely in line with the *in vivo* result that DOX+EGCG showed the best result in suppression p-NF-κB and p-MDM2, and increasing p53, which suggested that EGCG could work synergistically with DOX as an adjuvant reagent. EGCG was the most abundant and bioactive polyphenol from green tea, which showed efficacies in cancer prevention and treatment of tumors, as an efficient natural preventive agent or adjuvant sensitizer in combination therapy (Kallifatidis et al., 2016). We previously found that EGCG suppressed the bladder cancer SW780 tumor growth by down regulation of NF-κB (Luo et al., 2017). Moreover, it was demonstrated that NF-κB could promote the expression of oncogene MDM2, which in turn inhibit the activation of p53 (Zhuang et al., 2014). Jin et al. found that EGCG promoted p53 accumulation and activity via inhibition of MDM2 in human lung cancer cells (Jin et al., 2013). Same findings were also shown in human prostate carcinoma LNCaP cells that EGCG induced apoptosis via stabilization of p53, downregulation of MDM2 protein, and negative regulation of NF-κB activity, thereby decreasing the expression of Bcl-2 (Hastak et al., 2003). Our results demonstrated that EGCG was effective in suppressing the phosphorylation of NF-κB, inhibiting the expression of MDM2, and activating the downstream proteins of Bcl-2 and PARP, and then promoted the apoptosis induction, which might contribute to the synergistic effect of combination with DOX against bladder cancer proliferation and migration. In addition, DOX up-regulated the expression of p53, worked together with EGCG, and then amplified the anti-tumor effect in bladder cancer via NF-κB/MDM2/p53 pathway. Once NF-κB was inhibited in bladder cancer cells, the expression of p-MDM2 was decreased and p53 was up-regulated, and the combination of DOX+EGCG showed no obvious effect in SW780 cell migration and proliferation (Figure 6), indicating the importance of NF-κB/MDM2/p53 pathway played in DOX+EGCG induced effects.

Our results have clearly demonstrated that the combination of DOX and EGCG worked together in decreasing the phosphorylation of NF-κB and MDM2, and increasing the expression of p53, and showed significant anti-proliferation and anti-migration effects both *in vitro* and *in vivo*. This finding sheds light on the combination of DOX+EGCG on tumor inhibition in bladder cancer, and provides clear directions for mechanism study. Besides, the effective dose of EGCG worked synergistically with DOX and enhanced the apoptosis induction and anti-migration effects of DOX without obvious cytotoxic to cells, indicating that EGCG could be a safe and natural supplement for bladder cancer prevention and treatment in combined with DOX. In addition, EGCG also got other comprehensive benefits, such as anti-oxidation, cardiovascular protective effects, which made EGCG a good candidate for combination therapy in cancer treatment and comprehensive health care.

In conclusion, our results present the first evidence on the anti-tumor effect of DOX and EGCG against bladder cancer via NF-κB/MDM2/p53 pathway. More detailed molecular mechanisms, for instance, genomic and proteomic responses underlying the DOX+EGCG–induced bladder cancer cell apoptosis and anti-metastasis remain to be elucidated. Besides, further investigation on primary cultured bladder cancer cells, patient-derived xenografts model or clinical investigations were needed to determine the clinical efficacy of EGCG in combination with DOX. Our observation holds promise for further studies to develop EGCG as a potential anti-tumor adjuvant in combination with DOX against bladder cancer.

## Data Availability Statement

The raw data supporting the conclusions of this article will be made available by the authors, without undue reservation.

## Ethics Statement

The animal study was reviewed and approved by Animal Ethics Committee of Shenzhen Second People's Hospital.

## Author Contributions

K-WL provided the idea and wrote the manuscript. K-WL, X-hZ, TZ, JZ, and H-cG involved in cell culture, flow cytometry experiments, western blot, transwell migration assay, animal study, and immunochemistry assays. X-LL and W-RH designed the work and revised the manuscript. All authors contributed to the article and approved the submitted version.

## Conflict of Interest

The authors declare that the research was conducted in the absence of any commercial or financial relationships that could be construed as a potential conflict of interest.
